# Augmenting Clinical Interventions in Psychiatric Disorders: Systematic Review and Update on Nutrition

**DOI:** 10.3389/fpsyt.2021.565583

**Published:** 2021-05-05

**Authors:** Samuel J. Offor, Chinna N. Orish, Chiara Frazzoli, Orish E. Orisakwe

**Affiliations:** ^1^Department of Pharmacology and Toxicology, Faculty of Pharmacy, University of Uyo, Uyo, Nigeria; ^2^Department of Anatomy, Faculty of Basic Medical Sciences, College of Health Sciences, University of Port Harcourt, Port Harcourt, Nigeria; ^3^Department of Cardiovascular and Endocrine-Metabolic Diseases, and Aging, Istituto Superiore di Sanità, Rome, Italy; ^4^Department of Experimental Pharmacology & Toxicology, Faculty of Pharmacy, University of Port Harcourt, Port Harcourt, Nigeria; ^5^African Centre of Excellence for Public Health and Toxicological Research (ACE-PUTOR), University of Port Harcourt, Port Harcourt, Nigeria

**Keywords:** psychiatry, mental disorder, microbiome, biomarker, probiotics, nutrition, food

## Abstract

There is a strong relationship between a healthy diet and mental well-being. Several foods and food compounds are known to modulate biomarkers and molecular mechanisms involved in the aetiogenesis of several mental disorders, and this can be useful in containing the disease progression, including its prophylaxis. This is an updated systematic review of the literature to justify the inclusion and recognition of nutrition in the management of psychiatric illnesses. Such foods and their compounds include dietary flavanols from fruits and vegetables, notable antioxidant and anti-inflammatory agents, probiotics (fermented foods) known to protect good gut bacteria, foods rich in polyunsaturated fatty acids (e.g., Omega-3), and avoiding diets high in saturated fats and refined sugars among others. While the exact mechanism(s) of mitigation of many nutritional interventions are yet to be fully understood, the evidence-based approach warrants the inclusion and co-recognition of nutrition in the management of psychiatric illnesses. For the greater public health benefit, there is a need for policy advocacy aimed at bridging the knowledge gap and encouraging the integration of nutritional intervention with contemporary therapies in clinical settings, as deficiencies of certain nutrients make therapy difficult even with appropriate medication.

## Introduction

Mental disorders are widespread and impact significantly on health (1). In 2016, mental and addictive disorders affected more than 1 billion people globally and contributed 7% of the global burden of diseases (2). However, mental disorders manifest differently; according to WHO, they are generally characterized by a combination of abnormal thoughts, perceptions, emotions, behavior, and relationships with others (1). Current treatment involves the use of drugs such as antidepressants, antipsychotics, sedative-hypnotics, anxiolytics, stimulants, and mood stabilizers, along with psychotherapy (talk therapy). Electroconvulsive therapy (ECT) involving the application of electrical currents to the brain is used in some disorders that are unresponsive to other treatments.

“Nutritional psychiatry” pivots on the impact of nutrition (food) on the state of mind and mood. This presents an opportunity to augment clinical interventions as well as to mitigate the adverse effects of medications used in the treatment of psychiatric disorders (3, 4). Accumulating literature suggests a significant relationship between poor diet and the exacerbation of mood disorders, such as anxiety, depression, and other neuropsychiatric conditions (5). The likelihood of a healthy diet to produce beneficial effects on mental health among clinical and non-clinical subjects deserves more attention (6), and dietary interventions need to be refined and scaled up for maximum benefit in the management of mental disorders (7–9).

This review aims at updating the concept of “Nutritional Psychiatry” by (i) highlighting the various biomarkers and molecular mechanisms that form the hallmark of various mental disorders; (ii) examining foods and food compounds that can ameliorate the mechanistic derangement as evidence for the possibility of incorporating dietary interventions in the clinical management of psychiatric disorders.

## Methodology

Multiple online searches were carried out in the databases of Medline, Pubmed, Scopus and Google Scholar in May 2020 using terms like “Nutritional psychiatry,” “food and mental health,” “diets in psychiatry,” “nutrition and mental disorders,” “food and food compounds and mental health,” “biomarkers of psychiatric disorders,” and “mechanisms of mental disorders.” Sourced works of literature were screened, and full texts were obtained. Inclusion and exclusion criteria determined the suitability of the literature used in this review. In particular, studies were included if focusing on a whole food, supplements, or compounds (isolated from food) targeting psychiatric disorders. Articles were excluded when (a) not relevant, i.e., the nutritional source was meant to mitigate illnesses different from mental disorders, (b) unavailable in English, and (c) unavailable in full-text.

## Results and Discussion

### Search Results

**Figure 1:**
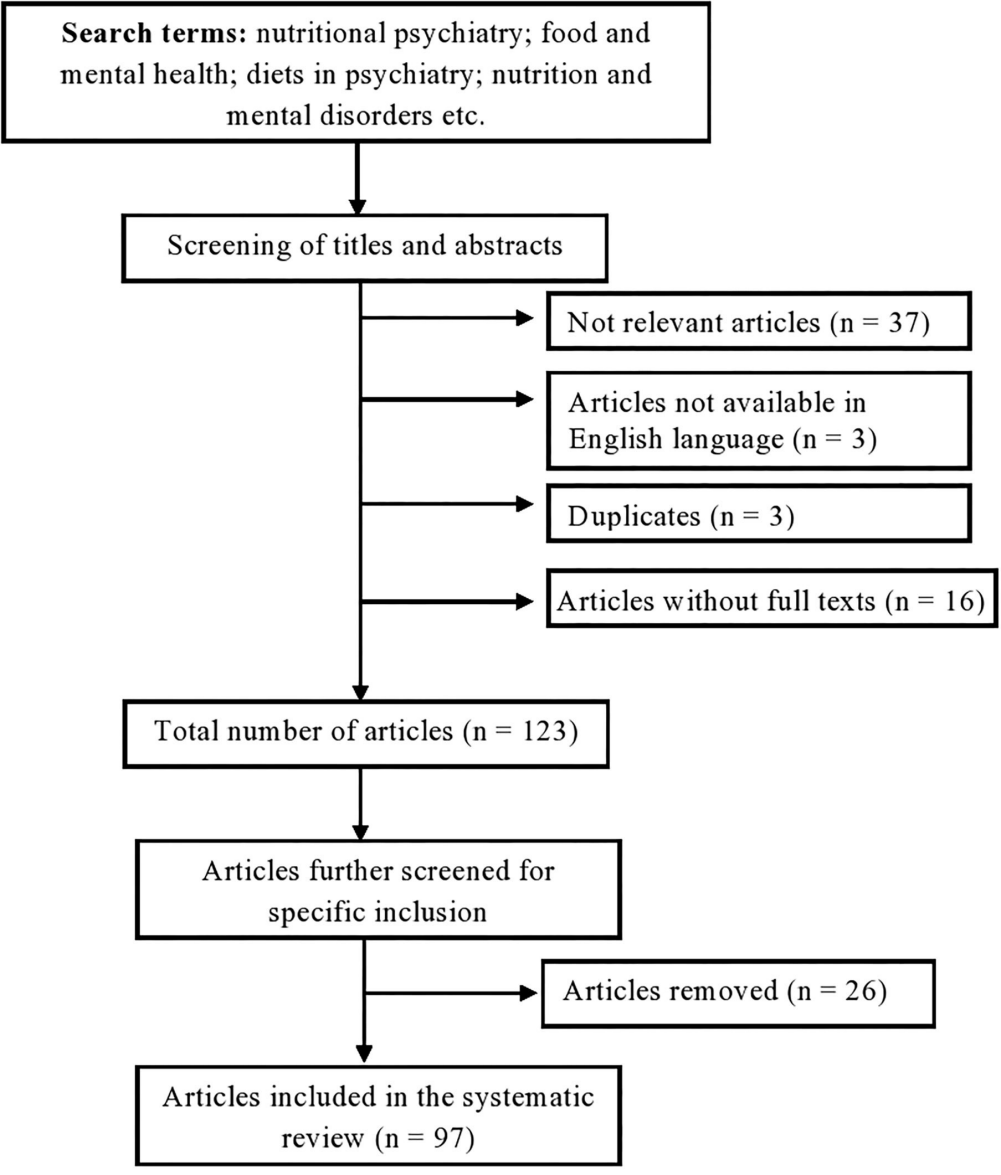
Flow diagram of search selection PRISMA.

One hundred and eighty-two (182) studies were found in the initial search. After a screening of both titles and abstracts, 59 articles were excluded; in particular, 37 articles were not relevant, 16 full texts were unavailable, 3 were unavailable in English, 3 were duplicates. Further review of the full texts of the remaining 123 articles with strict application of the inclusion and exclusion criteria resulted in the exclusion of 26 articles, thus leaving 97 studies that were included in this review (Figure 1).

There are several types of diagnosable mental disorders that are known to cause significant alterations in behavioral, thoughts, emotional and functional disabilities. They include but are not limited to the following: depression (1), bipolar disorder (10), schizophrenia (11), dementia, autism spectrum disorder, generalized anxiety disorder (12, 13), attention-deficit/hyperactivity disorder (14), obsessive-compulsive disorder (15, 16), post-traumatic stress disorder (17) and eating disorders such as anorexia nervosa and bulimia nervosa (18).

## Molecular Mechanisms and Biomarkers of Psychiatric Disorders

The term biomarker can be referred to as a characteristic that is objectively measured and evaluated as an indicator of normal biologic processes, pathologic processes, or biological responses to a therapeutic intervention (19). It can be a gene, a group of genes, proteins, or other biomolecules (20). Due to the complexity of psychiatric disorders, biomarkers cannot be limited to molecular biology in psychiatry. Advances in neuroimaging methods have modernized the understanding of the bio-clinical substrata of many psychiatric disorders (21, 22). Clinical uses of biomarkers in psychiatry involve measuring them before the intervention and with the goal of predicting drug response, diagnosis, therapeutic failure, prognosis, pharmacotoxicity, and classification within diagnostic categories (23–26). They include inflammatory biomarkers such as high levels of cytokines and C-reactive protein (CRP), changes in serum molecules involved in pro-inflammatory and oxidative stress response, including hyperactivation of the hypothalamic-pituitary-adrenal (HPA) axis (27). Elevated levels of pro-inflammatory cytokines have been observed in patients with depression (28–31), schizophrenia (32, 33), and eating disorders (34).

Protein biomarkers involve the expression of proteins in the brains such as growth differentiation factor-15, hemopexin, hepsin, matrix metalloproteinase-7, retinol-binding protein-4, and trans-thyretin, which have been reported as biomarkers to distinguish patients with bipolar disorder from those without the disorder (35); up-regulation of microRNA utilized as a biomarker for diagnosis of patients with schizophrenia (36); increased cerebrospinal fluid levels of β-amyloid, tau, and phosphor-tau for Alzheimer's disease (37).

Disturbances in central and peripheral Neurotransmitters biomarkers are also indicators of mental disorders such as major depressive disorders. These neurotransmitters include dopamine, glutamate, γ-aminobutyric acid (GABA), and serotonin (38). Neurotrophic biomarkers such as expression of the brain-derived neurotrophic factor, BDNF in cognitive impairments in individuals with mental disorders is of utmost research interest (39). Electrophysiological biomarkers used in psychiatry include imbalances in resting heart rate (RHR), heart rate variability (HRV), respiration rate (RR), skin temperature (ST), skin conductance (SC) (40, 41), event-related potentials (ERP) and visual evoked potentials (42). ERP measures the electrical activity of the cerebral surface that represents a distinct phase of cortical processing. It is made up of two components, namely P300 positivity and N200 negativity (43). It has been reported that P300 activity may serve as a useful biomarker of attention and as a screen for combination-drug therapy in investigations of anti-Alzheimer drugs (44). In addition, several neuroimaging techniques like Magnetic Resonance Imaging (MRI), Positron Emission Tomography scan (PET scan), Single Positron Emission Tomography scan (SPECT scan), Magnetic [Resonance Spectroscopy (MRS), Functional Magnetic Resonance Imaging (fMRI), and Diffuse Tensor Imaging (DTI) are currently employed to find biomarkers for mental illness (45) and to clearly elucidate the neural basis of the psychiatric disorder (40)]. The gut microbiota could control functional pathways in the brain and, therefore, useful as both biomarkers and potential drug targets in mental disorders (46). The gut microbiome has been demonstrated to play an essential role in the development and function of the hypothalamic-pituitary-adrenal (HPA) axis, which mediates the stress response and is involved in a range of psychiatric disorders, especially depression and anxiety disorders (47, 48). The use of antibiotics, Western diets, and excessive-stress lifestyles culminate in gut bacterial imbalances, known as dysbiosis, in addition to low diversity. Bacteria have the ability to produce GABA, tryptophan, 5-HT, and several neurotransmitters and monoamines MOA. Pathophysiology of mental disorders has also been linked to bacterial translocation via increased gut permeability (49). Anxiety, stress, and depression can increase gut barrier permeability, resulting in a ‘leaky gut,’ which allows bacteria to seep into circulation, leading to the inflammatory response (50–52).

**Figure 2:**
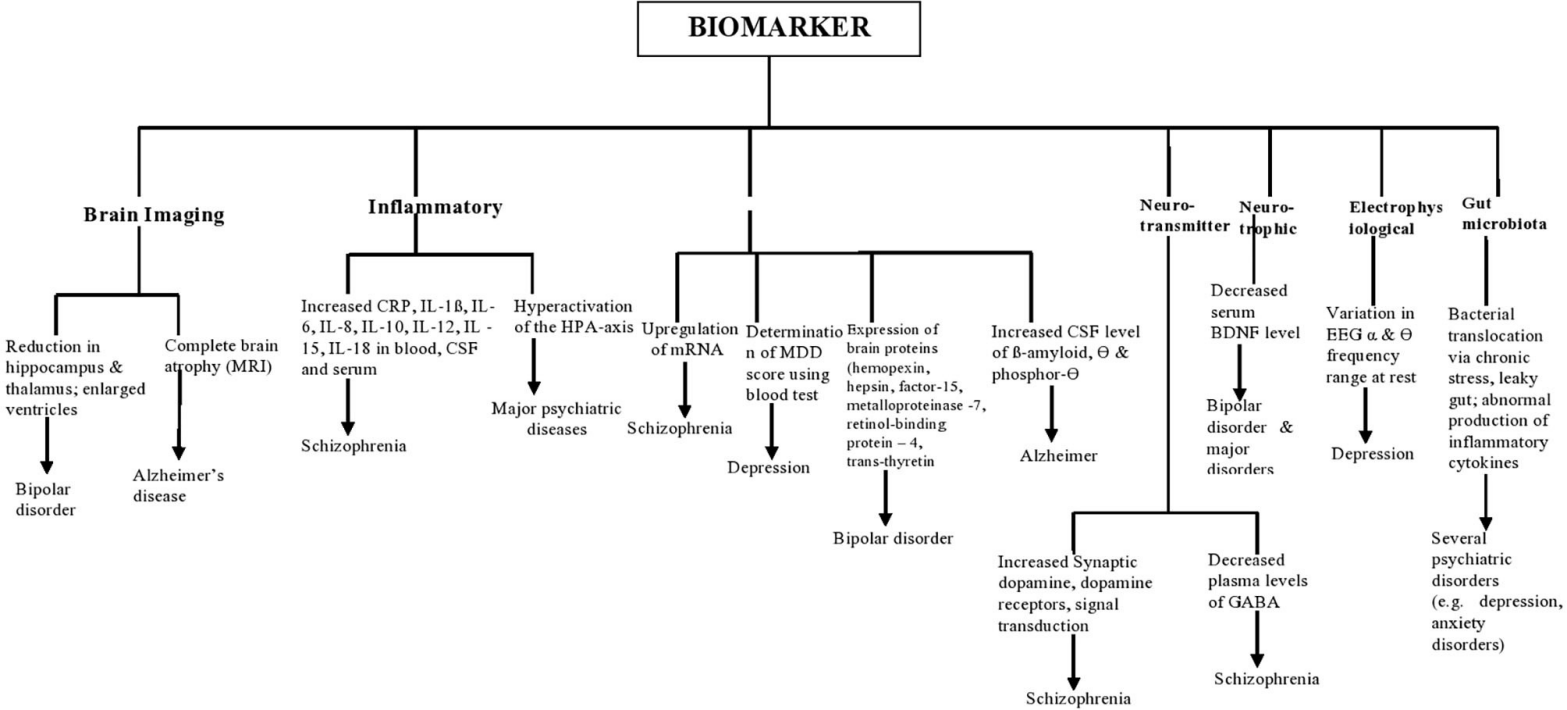
Schematic illustration of biomarkers in some psychiatric disorders.

A schematic illustration of some biomarkers in psychiatric disorders is shown in Figure 2.

## Foods and Food Compounds that Affect Psychiatric Biomarkers

A strong relationship between a healthy diet and mental well-being is often reported by people. Elation, mental health, and well-being have reportedly been increased by the increase in the consumption of fresh fruits and vegetables (53–56). Dietary flavanols, namely kaempferol, isorhamnetin, and myricetin (i.e., components of many fruits, vegetables, and tea), have been linked to a significantly lower risk of development of Alzheimer's disease (57). Many flavonoid classes, including flavonols, are anti-inflammatory and antioxidants.

Some common fruits like citrus (e.g., lime, lemon, orange, tangerine, grape), guava, cashew, mango, pawpaw, pineapple, avocado, banana, African star apple, sweetsop/ sugar apple, breadfruit, soursop, African bush mango, passion fruit, apple, dates (58) and vegetables (e.g., tomatoes, okra, eggplant, cucumber, beets, garlic, onion, and ginger) found in sub-Saharan Africa may be of nutritional psychiatry relevance (58). These fruits and vegetables have high levels of micronutrients such as zinc, magnesium, selenium, iron, and vitamins (59, 60). These micronutrients may modulate the risk of mental disorder, such as depression, via effects on the production and activity of monoamine neurotransmitters like serotonin, alterations to the HPA system, glutamatergic signaling, or inflammatory and oxidative stress (61, 62). These plant-based foods contain antioxidant phytochemicals, such as polyphenols, vitamin C, and flavonoids, i.e., substances whose antidepressant-like or anxiolytic effects have been reported (63, 64).

**Figure 3:**
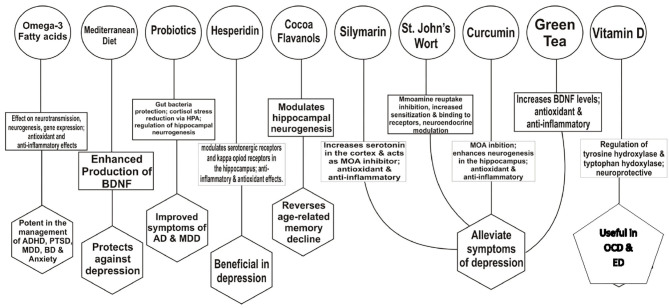
Bio-molecular mechanisms of dietary interventions in some mental disorders. HPA, hypothalamic-pituitary-adrenal axis; OCD, Obsessive-Compulsive Disorder; MOA, Monoamine oxidase; BD, Bipolar disorder; BDNF, brain-derived neurotrophic factor; MDD, Major Depressive Disorder; ADHD, Attention deficit hyperactivity disorder; PTSD, Post traumatic stress disorder; AD, Alzheimer dementia; ED, Eating disorders.

Several foods/food compounds are known to affect some psychiatric disorders in different ways. They include several phytochemicals like flavonoids, probiotics, omega-3 fatty acids, vitamins, myoinositol, Curcumin, plant parts like valerian root, milk thistle, and green tea (Table 1). These nutrients may affect mental disorders via several mechanisms such as the production and activity of monoamine neurotransmitters, neurotransmission, modulation of hippocampal neurogenesis, hypothalamic-pituitary-adrenal (HPA) system, anti-inflammatory and antioxidant effects, augmenting the production of brain-derived neurotrophic factor, BDNF or the protection of gut bacteria, among others. These bio-molecular mechanisms of dietary interventions in some mental disorders are summarized in Figure 3.

**Table 1:** Some] foods/food compounds that affect psychiatric disorders.

**Food or food compounds**	**Comment**	**References**
Dietary flavanols (kampferol, isorhamnetin, myricetin)	They are components of many fruits, vegetables and tea. They lower risk of development of Alzheimer's disease	(57)
Hesperidin (citrus-derived flavonoid)	In depression: (i) Modulation of serotonergic 5-HT1A receptors and kappa opioid receptors in the hippocampus (ii) Anti-inflammatory and antioxidant effects.	(65–67)
Cocoa flavanols	Reverses age-related memory decline through modulation of hippocampal neurogenesis	(68, 69)
Mediterranean diet (whole grains, sea food, poultry, legumes, beans nuts, fresh fruits, leafy vegetables, healthy fats, and less red meat)	Protection against depression linked to enhanced production of brain-derived neurotrophic factor (BDNF)	(3, 70)
Probiotics (e.g., fermented foods such as yogurt active cultures)	(i) They protect gut bacteria; reduce cortisol stress via HPA; regulate hippocampal neurogenesis. (ii) Traditional African fermented foods contain live organisms capable of producing health-promoting compound and can act as probiotic strains.	(3, 40, 71) (72, 73)
Omega-3 fatty acids (from fish, seafood, grass-fed beef)	Effective in the treatment of ADHD, PTSD, major depressive disorder, bipolar depression. They affect neurotransmission, neurogenesis, gene expression and have antioxidants and anti-inflammatory properties.	(74–76)
Myoinositol	• An endogenous isomer of glucose; also present in nuts, grains, beans and fruits. Effective in treatment of OCD • Mechanism of action may involve modulating the reuptake of serotorin and increasing 5-HT2 receptor density.	(77–79)
Silymarin	• A flavonoid from the plant, Milk thistle. It has anti-inflammatory, antioxidant, antidepressant effects. • Increases, serotonin in the cortex and acts as a monoamine oxidase inhibitor	(80, 81)
Milk thistle	It has similar effects with fluoxetine in therapy of OCD but without severe adverse effects.	(82)
Valerian root (Valeriana officialis L)	Contains (i) aleuronic acid that is associated with modulation of GABA receptors (ii) Valepotriates, reported to be effective in treatment of psychotic symptoms of severe anxiety.	(83, 84)
St. John's Wort (*Hypericum perforatum*)	• It contains flavonoid. Its activity involves monoamine reuptake inhibition, neuroendocrine modulation, increased sensitization and binding to receptors (e.g., 5-HT). • It is equivalent to antidepressant in the treatment of depression	(85, 86)
Vitamins	Vitamin D: deficiency may affect OCD etiology by affecting the pathway of serotonin and catecholamine synthesis. It does this through regulation of the enzymes, tyrosine hydroxylase and tryptophan hydroxylase in addition to its neuroprotective effects. Vitamin B12: Deficiency causes depression, mania, psychosis Vitamin B9 (Folic Acid): deficiency result *in utero*- neurodevelopmental defects and is linked with depression in adults. Vitamin B3 (niacin): Deficiency causes pellagra with resultant dementia	(87–92)
Curcumin	A polyphenol obtained from tumeric plant. It reduces symptoms of depression.	(93, 94)
Epigallocatechin gallate (EGCG)	A polyphenol found in Green tea. It alleviates symptoms of stress and depression	(93)

High doses of naturally occurring cocoa flavanols have been reported to reverse memory decline associated with age (68). Flavanols seem to selectively improve the function of the dentate gyrus, i.e., a region within the hippocampus that is associated with aging and age-related memory decline (68). The hippocampus is a region of the brain associated with memory, learning, and mood. The extent of neurogenesis in the hippocampus is directly related to cognition and mood. Modulation of hippocampal neurogenesis by diet is a possible mechanism by which nutrition affects brain function, plasticity, and mental health (69).

Hesperidin, i.e., a citrus-derived flavonoid, has been reported to have neuroprotective effects, particularly against depression, learning, and memory deficits (95–97). Possible mechanisms of its antidepressant-like effects are regulation of serotonergic 5-HT1A receptors (65) and kappa opioid receptors in the hippocampus (66). Hesperidin has both anti-inflammatory and antioxidant effects (67). In a model of aluminum chloride-induced neuroinflammation in the hippocampus, the anti-inflammatory properties of hesperidin involve a reduction in the levels of pro-inflammatory mediators like tumor necrosis factor α (TNF-α) (98). Hesperidin has also been shown to protect the hippocampus by reducing levels of nitrate/nitrite while increasing levels of BDNF in the mouse (99). Its free radical scavenging and antioxidant abilities tend to ameliorate the shortfalls in the activity of glutathione peroxidase, glutathione reductase, catalase, and superoxide dismutase. In experimental models of stroke, irradiation, and LPS-induced endotoxicity, these antioxidant enzymes are down-regulated in the brain (100–102). The Mediterranean diet involves eating whole grains, seafood, and poultry (at least twice a week) and consuming legumes, beans, fresh fruit, leafy green vegetables, nuts (almonds, walnuts), cruciferous vegetables (cauliflower, broccoli), healthy fats (olive and canola oil), and a limited amount of red meat (3). According to a recent study, a Mediterranean diet and avoiding inflammation-producing foods may protect against depression (103). The mechanisms involved may be linked to enhanced production of BDNF, and therefore important functions such as neuroplasticity, neuronal survival, as well as growth and differentiation of new neurons and synapses (70). Low serum BDNF levels have been found in a number of psychiatric disorders like schizophrenia, major depressive disorder, PTSD, and Alzheimer's dementia (104). Diet can regulate or dysregulate the gut microbiome. Healthy gut microbiota is central in the regulation of serotonin metabolism because at least 90% of serotonin receptors are located in the gut (105). Alteration in the balance between “good” and “bad” bacteria may result in several diseases, including mood and cognitive disorders. Probiotic-rich foods (e.g., fermented foods such as yogurt with active cultures) are known to protect good gut bacteria (3). Fermented foods contain strains of Lactobacillus as well as yeasts and are vital because they contain both probiotic microbiota and microbial metabolites (59). Prebiotics, in their turns, include non-digestible fiber, which stimulates the growth of probiotics (70). Many prebiotics and probiotics reduce cortisol stress in healthy subjects (71, 106, 107). Several studies suggest that both individuals with clinically diagnosed cases and healthy individuals experiencing some anxiety and mood disorders benefit from the consumption of probiotics (108, 109). Several fermented foods are traditionally used in different parts of Africa' (110). These include fermented non-alcoholic cereals (mainly from sorghum, millet, and maize), starchy root crops (mainly from cassava), animal proteins (mainly dairy products), vegetable proteins (from legumes and oilseeds), and alcoholic beverages (e.g., from cereals, sap, honey, fruits) (111). These traditional African probiotics contain live microorganisms capable of producing health-promoting compounds like antimicrobials and essential nutrients or molecules with antioxidant activity (72, 73). The western diet, known for its content of ultra-processed foods, has been reported to change microbiome (gut environment), leading to reduced Lactobacilli (112), gut inflammation, and possibly contribute to disorders (113). Mediterranean diet reduces the numbers of inflammatory/pathogenic bacteria like Escherichia coli and increases important commensal bacteria such as Bifidobacteria (114), Clostridium cluster XVIa, and Faecalibacterium prausnitzii (115). Vegetarian diets have been reported to alter the microbial composition and reduce inflammation of the gut (116, 117). Pathogenesis of psychosis has been linked to anomalies in glucose tolerance, insulin resistance, mitochondrial dysfunction, and energy metabolism disturbances. These could be potential mechanisms for the effect of a ketogenic diet. This diet, high in fat, and low in carbohydrate, utilize ketone bodies as the fuel source for the brain, instead of glucose (118, 119). A report from investigators with the Nutrition Network of the European College of Neuropsychopharmacology (ECNP) postulates that a ketogenic diet may decrease seizures in children with epilepsy (5, 120).

Some diets have potentially harmful effects on the brain. A diet high in saturated fats and refined sugars has a powerful negative impact on brain proteins (neutrophins). Neutrophins are very important in depression: they protect the brain against oxidative stress and promote the growth of new brain cells (121). Del-Ponte and co-workers reported recently that food high in refined sugar and saturated fat might cause an increased risk for hyperactivity (ADHD) compared to fruits and vegetables (122). Eliminating the underlying suspected trigger foods may work as secondary prevention of food-induced ADHD: the “few-foods approach” is a diagnostic protocol allowing to determine whether or not individually composed few food diets (one food per week is added to the diet) are a trigger of ADHD. If the behavioral problems do not recur, the food can be included in the diet without restriction (123, 124).

Foods that contain aspartame, a food additive, are forbidden for people with phenylketonuria (a birth defect that causes the amino acid phenylalanine to build up in the body) as this can result in brain damage, intellectual disabilities, behavioral symptoms, or seizures (125).

Several studies have corroborated the fact that deficiency of some vitamins and other essential nutrients lead to cognitive impairments (126, 127). Vitamin D plays an important role in immunity modulation, inflammatory response, and antioxidant processes, as well as in normal brain development and functioning, neurotransmission, neuroprotection, proliferation, and differentiation (80, 128, 129). Vitamin D deficiency can be associated with numerous neuropsychiatric diseases, including autism, major depressive disorder, schizophrenia, and Obsessive-Compulsive Disorder (OCD) (130, 131). Vitamin D deficiency may contribute to OCD etiology by (i) affecting the pathway of serotonin and catecholamines synthesis, (ii) regulation of the levels of the enzymes tyrosine hydroxylase and tryptophan hydroxylase, (iii) deprived neuroprotective effect (87, 88). In adults aged 65 years and above, higher vitamin D serum levels were associated with better attention and working memory performance (132). Vitamin D has also been reported to support the nervous system and brain functions such as impulsive behaviors, known to be of importance in the prognosis and treatment of patients with Eating Disorders (133). Vitamin B12 deficiency causes depression, lethargy, poor memory, fatigue, mania, and psychosis (89), while vitamin B3 (niacin) deficiency causes pellagra with resultant dementia (90). Deficiency of vitamin B1 (thiamine) causes beriberi and numbness as CNS symptoms, while vitamin B9 (folic acid) deficiency results in *in utero* neurodevelopmental defects and is linked with depression in adults (91, 92).

Foods rich in polyunsaturated fatty acids, PUFAs (e.g., Omega-3s), and polyphenols have also been reported to have beneficial effects in neuroinflammation, cognitive performance, mood, and stress reactivity (134–137). Omega-3 fatty acids are effective in the treatment of attention-deficit/ADHD, major depressive disorder, bipolar depression, and post-traumatic stress disorder, or PTSD (74, 75). Omega-3 fatty acids are found in fish, seafood, and grass-fed beef (70). Omega-3 fatty acids are an integral part of neuronal cell membranes and affect several physiological mechanisms in the central nervous system. They affect neurotransmission, gene expression, neurogenesis, neuronal survival and also have antioxidants and anti-inflammatory properties (76). A balance between omega-6 and omega-3 fatty acids seems to be relevant in some mental disorders, as high omega-6 to an omega-3 fatty acid ratio in the blood has been associated with major depressive disorder and ADHD (56, 70).

Myoinositol (MI), an endogenous isomer of glucose also present in nuts, grains, beans, and fruits, is used in the treatment of mental disorders. It is essential for the synthesis of membrane phospholipids and for the intracellular secondary messenger cycle (77). Although some studies found no evidence for the efficacy of myoinositol in OCD treatment, others have reported the effectiveness of myoinositol supplementation in the treatment of OCD (78, 138). Available clinical evidence suggests that MI may potentially be effective as monotherapy in OCD (80). The suggested mechanisms of action involve modulation of the reuptake of serotonin and an increase in 5-HT2 receptor density (79). Silymarin, a flavonoid derived from the plant Milk thistle (Silybummarianum), has been reported to have anti-inflammatory, antioxidant, immune modulator, sedative, and antidepressant effects (80). It increases serotonin in the cortex and acts as a monoamine oxidase inhibitor (81). The effect of milk thistle and fluoxetine are alike in the treatment of OCD, and their positive effect starts in the 5th week without severe adverse effects (82). Valerian root (obtained from the plant Valeriana officinalis L contains aleuronic acid associated with the modulation of GABA receptors) (83) and valepotriates (effective in the treatment of the psychotic symptoms of severe anxiety) (84). St John's Wort (*Hypericum perforatum*), which is of plant origin, has been reported to be equivalent to an antidepressant in the treatment of depression (85, 86). It contains flavonoids, and its neurobiological activity involves monoamine reuptake inhibition, neuroendocrine modulation, increased sensitization, and binding to receptors (e.g., 5-HT) (85). Although some herbal medicines may provide a synergistic effect with conventional drugs, there should be some precautions in the use of some herbal supplements and some pharmaceuticals, for example, St John's Wort with SSRIs due to potential adverse serotonin syndrome (80). Polyphenols are natural compounds present in plant-based foods. They have unique properties and are capable of combatting oxidative stress as well as stimulate the activation of molecules that aid in synaptic plasticity, thereby enhancing cognitive function (93). Notable examples of polyphenols include Epigallocatechin gallate (EGCG) from green tea and Curcumin from turmeric. Apart from their antioxidant and anti-inflammatory properties, their mechanisms of action involve increased expression of BDNF, which enhances the reversal of neuronal atrophy and behavioral deficits (139). Curcumin has been reported to mitigate symptoms of depression by enhancing neurogenesis in the hippocampus and frontal cortex (94). It also inhibits the action of monoamine oxidase enzymes, thus preventing the breakdown of monoaminergic neurotransmitters, thereby increasing serotonin and dopamine levels (140). Epigallocatechin gallate from green tea has been reported to alleviate symptoms of stress and depression (93).

## Conclusion

The field of nutritional psychiatry though still new, is currently undergoing intensive research, resulting in several positive research findings. As with many other diseases, several foods and food compounds are known to modulate biomarkers and molecular mechanisms involved in the aetiogenesis of several mental disorders, and this can be useful in containing the disease progression, including its prophylaxis. While the exact mechanism(s) of mitigation of many nutritional interventions are yet to be fully understood, the evidence-based approach warrants the inclusion and co-recognition of nutrition in the management of psychiatric illnesses. For the greater public health benefit, there is a need to advocate for policies aimed at bridging the knowledge gap and encourage the utilization and integration of nutrition in addition to contemporary therapies in clinical settings, as deficiencies of certain nutrients make therapy difficult even with the right medication. This is especially advantageous in developing, resource-challenged nations laden with inadequate healthcare funding for mental disorders, despite the condition being rife in the region and given the fact that these food substances are affordable and readily available in these nations.

## Data Availability Statement

The original contributions presented in the study are included in the article/supplementary material, further inquiries can be directed to the corresponding author/s.

## Author Contributions

SO conducted the search, data extraction, and drafting of manuscript. CO and OO conceptualization, reviewed the draft manuscript, and certified final manuscript. CF reviewed the draft manuscript. All authors contributed to the article and approved the submitted version.

## Conflict of Interest

The authors declare that the research was conducted in the absence of any commercial or financial relationships that could be construed as a potential conflict of interest.
